# How Expert Advice Influences Decision Making

**DOI:** 10.1371/journal.pone.0049748

**Published:** 2012-11-21

**Authors:** Dar Meshi, Guido Biele, Christoph W. Korn, Hauke R. Heekeren

**Affiliations:** 1 Berlin School of Mind and Brain, Humboldt Universitaet zu Berlin, Germany; 2 Department of Education and Psychology, Freie Universitaet Berlin, Germany; 3 Dahlem Institute for the Neuroimaging of Emotion, Freie Universitaet Berlin, Germany; 4 Center for the Study of Human Cognition, Department of Psychology, University of Oslo, Norway; Brain and Spine Institute (ICM), France

## Abstract

People often use expert advice when making decisions in our society, but how we are influenced by this advice has yet to be understood. To address this, using functional magnetic resonance imaging, we provided expert and novice advice to participants during an estimation task. Participants reported that they valued expert advice more than novice advice, and activity in the ventral striatum correlated with this valuation, even before decisions with the advice were made. When using advice, participants compared their initial opinion to their advisor’s opinion. This comparison, termed the “opinion difference”, influenced advice utilization and was represented in reward-sensitive brain regions. Finally, the left lateral orbitofrontal cortex integrated both the size of the opinion difference and the advisor’s level of expertise, and average activity in this area correlated with mean advice utilization across participants. Taken together, these findings provide neural evidence for how advice engenders behavioral change during the decision-making process.

## Introduction

Many important decisions are made while under the influence of expert advice, from a politician receiving counsel when deciding whether to raise taxes, to a cancer patient being advised by their doctor when deciding whether to undergo chemotherapy. Advice is also valuable to us; we give it such high regard that billions of dollars change hands every year to receive counsel [1]. These recommendations could come in the form of guidance on corporate strategy from a top consulting firm, or suggestions on personal money management from a financial expert. Furthermore, in the real world the level of expertise of advisors varies; decision makers encounter people with in-depth knowledge who can provide high quality advice, as well as less-informed people providing advice of a lesser quality. With advice taking playing such an important role in our society, especially the role of expert advice in our economy, surprisingly little is understood about how we integrate, and are influenced by, information from advisors with different levels of expertise.

Here, we conceptualized advice-taking as consisting of three cognitive processes: (1) the valuation of advice, (2) the assessment of the “opinion difference” (i.e., the comparison between an advisor’s opinion and one’s own opinion), and (3) the process of combining valuation and the opinion difference resulting in actual advice utilization. Regarding the first process, the valuation of advice, it is well-established that people use advice from experts to a greater degree than advice from novices [2]–[4]. One possible explanation for the strong influence of expert advice is that people value it more than novice advice, even before they actually make a decision and discover the outcome of this decision. Therefore, we hypothesized that the same brain areas that represent value when receiving money and objects, such as the ventral striatum and orbitofrontal cortex [5]–[7], also represent value when people discover they will be receiving either expert or novice advice.

Second, we hypothesized that when people receive an advisor’s opinion they compare it to their initial opinion. We call this comparison an “opinion difference”. The opinion difference is used in judging whether or not, or to what degree, a person is influenced by the advice [8]. For example, if an advisor’s opinion is similar to a person’s initial opinion, the opinion difference is low and the person uses the advice more. Conversely, if an advisor’s opinion disagrees with a person’s initial opinion, the opinion difference is high and the person is less influenced by the advice. Because advice is commonly used when making goal-oriented decisions where people try to obtain rewards or avoid punishments, we theorized that the change in neural activity due to the opinion difference occurs in previously established reward-sensitive areas such as the ventral striatum, amygdala, anterior cingulate gyrus, ventromedial prefrontal cortex and the orbitofrontal cortex [5]–[7], [9]–[11]. Our analysis of neuroimaging data reflected this *a priori* hypothesis (see Methods).

Finally, regarding the process of combining valuation and the opinion difference, we aimed to identify a region of the brain where neural activity represents the behavioral influence of advice. Activity in this area should fulfill two conditions. One, when a person receives advice, the neural signal within this area should reflect the consideration of the size of the opinion difference and the expertise level of the advisor relative to each other. For example, a person experiencing a large opinion difference when receiving advice from an expert may react differently compared to experiencing a large opinion difference when receiving advice from a novice. This difference in reaction when receiving expert versus novice advice may not hold when that person experiences a small opinion difference. In other words, there should be an interaction between the expertise level of an advisor and the size of the opinion difference. Two, the average activity in this brain area should correlate with the behavioral influence of advice at the individual level. Specifically, different people should perform this comparison to different degrees, which should result in individual differences in advice utilization.

There have been three previous neuroimaging studies concerning aspects of advice taking. These studies did not address the above-outlined cognitive processes, but rather focused on comparing decision making with or without advice, or examining neural activity when learning the outcome of decisions made with advice [12]–[14]. There have also been several neuroimaging studies which examined conformity to others, which is related to the social influence of explicit advice [15]–[17]. Furthermore, one study also examined the expertise of celebrities and its implicit influence on attitudes and memory of objects [18]. Here, we focused on the not yet investigated differences between utilizing explicit expert and novice advice, and on understanding brain activity at the time when people receive and utilize advice.

## Materials and Methods

### Ethics Statement

This study was approved by the local ethics committee at the Freie Universitaet Berlin, Germany. The study was carried out in accordance to the Declaration of Helsinki. Informed written consent was obtained from each subject before the study.

### Participants

We recruited 29 healthy, right-handed participants (12 male) between 20 and 30 years of age (mean = 23.3, SD = 2.8). All participants had no history of psychiatric or neurological disorder. German was the native language of all participants. Relevant to the experimental paradigm, no participant had previous experience renting apartments in New York City, or previous work experience in the real estate industry.

### Experimental Cover Story

Participants were told that they would be estimating the monthly rental price of apartments in New York City for a monetary reward. Furthermore, they were told that during the task they would encounter three different situations: 1. They would receive advice on the monthly rental price from an expert. This expert advice would be randomly selected from the suggestions for each apartment given by one of ten New York City real estate agents. 2. They would receive advice on the monthly rental price from a novice (“Laie” in German). This novice advice would be randomly selected from the suggestions for each apartment given by one of ten people similar to the participants, without experience in the New York City real estate market. Participants were told that these novices received the same training as the participants (see Procedure). Importantly, participants believed all advice was well-intentioned; they were told that the advisors, both experts and novices, were paid for how close their advice was to the real rental price. This removed the potentially confounding effect of trustworthiness on the expertise of advisors. 3. They would not receive advice. When participants encountered this no advice situation they were asked to “think again” about their estimate on the monthly rental price of an apartment. In order to motivate them to think again when not receiving advice, participants were told that research shows that people who think again and revise their initial opinion increase their accuracy [19].

Participants were paid 10 Euro for taking part in the study and were told that the accuracy of their estimates would be ascertained after the task. From the 120 trials, 8 would be randomly selected (4 from their first estimates and 4 from their second estimates, see Procedure) to be compared to the real rental price of the apartments. Participants were told they would receive 2 Euro if their estimate was within 100 Euro from the actual rental price, or 1 Euro if their estimate was between 101 and 300 Euro away from the actual price. Thus, participants had the potential to win an additional 16 Euro.

### Stimuli

Descriptions and price information of real apartments offered for rent in New York City were obtained from www.streeteasy.com (September 2010), a website which aggregates apartment listings. One hundred and thirty-three apartments (120 = experimental stimuli set, 10 = first training session set, 3 = second training session set; see Procedure) were selected as stimuli from a database of 6,062, and four attributes of each apartment were presented to participants in German: 1. Square meters (converted from square feet), 2. Number of rooms, 3. Number of bathrooms, and 4. Neighborhood. Neighborhood was an index of quality that ranged from 1 to 3. Neighborhoods were ordered according to average price per square meter and then divided into thirds. If the neighborhood of a specific apartment was in the lower third of the average price per square meter ranking, it was assigned to Neighborhood 1, if in the middle third it was placed into Neighborhood 2, and if in the top third it was placed into Neighborhood 3.

Apartments in the stimuli set ranged in price from 799 to 3188 Euro (mean = 1963.8, SD  = 612.8; converted from dollars), in square meters from 14 to 139 (mean = 60.2, SD = 22.7), in number of rooms from 1 to 6 (mean = 2.83, SD = 1.1), in number of bathrooms from 1 to 2 (mean = 1.1, SD = 0.3), and as mentioned above, in neighborhood index from 1 to 3 (mean = 1.98, SD = 0.8). A linear regression was performed on all 133 apartments used in the experiment and training sessions. All 4 attributes significantly predicted the monthly rental price of the apartment (F_(4,128)_ = 101.568, p<0.001; square meters p<0.001, number of rooms p = 0.013, bathrooms p = 0.007, neighborhood p<0.001).

In order for each participant to receive an even distribution of attributes and apartment values across each of the 3 experimental conditions (see Procedure), the 120 stimuli were divided into 3 groups of 40. Each group of apartments was then assigned to a single experimental condition for each participant, counterbalanced across participants. Between the three groups of apartments, there was no difference in apartment price (F_(2,117)_ = 0.474, p = 0.624), square meters (F_(2,117)_ = 0.105, p = 0.901), number of rooms (F_(2,117)_ = 0.132, p = 0.876), number of bathrooms (F_(2,117)_ = 0.084, p = 0.919), or neighborhood index (F_(2,117)_ = 0.051, p = 0.950).

### Procedure

Before scanning, each participant was given two training sessions. The first was to educate the participant on the actual prices of apartments in New York City, and the second was to acclimate them to the actual task procedure. In the first training session, which consisted of ten trials, participants saw a description of an apartment and were asked to estimate the monthly rental price. Participants were then shown the real rental price of the apartment as listed on streeteasy.com. In the second training session, participants practiced the actual experimental task for three trials, one for each experimental condition (expert advice, novice advice, no advice). All participants received the same apartments as stimuli for training presented in random order, and apartments used in the training sessions were not used as stimuli in the actual experiment.

**Figure 1:**
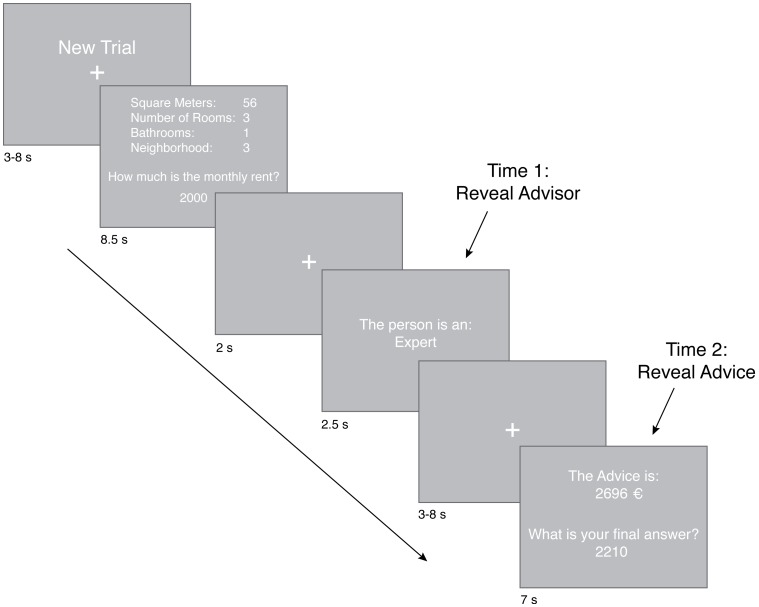
Experimental task. Participants had 8.5 s to estimate the real monthly rental price of an apartment in New York City by using four attributes (square meters, number of rooms, number of bathrooms, neighborhood). They then discovered the expertise level of their advisor (Time 1; 2.5 s). After a short delay, participants were then given advice on the rental price and allowed to adjust their answer (Time 2; 7 s).

The experimental task conducted in the MRI scanner is depicted in Figure 1. At the start of each trial participants were presented with a fixation cross, above which was written “New trial”. This lasted for 3 to 8 s (display times of this fixation cross followed an exponential distribution with most display times at the lower end of the range). Participants were next shown a description of a real New York City apartment and asked to estimate the monthly rental price within a time frame of 8.5 s. On the display in each trial, the starting amount of their estimate was 2000 Euro and participants used three buttons with a triangular spatial arrangement to adjust and confirm their estimate. They pressed left to decrease the amount, right to increase the amount, and the button at the top of the triangle to confirm their estimate. When the estimate was entered, a Euro symbol (€) appeared next to the amount. When the 8.5 s expired, participants saw a fixation cross for 2 s, followed by a display revealing who their advisor would be, if any. The display told them either: 1. The person is an expert, 2. The person is a novice, or 3. There will be no advice. This advisor information was revealed for 2.5 s, after which, another fixation cross was presented for 3 to 8 s (also following an exponential distribution). After this delay, participants had 7 s in which they were shown the advice amount, as well as the amount they entered as their first estimate, and allowed to make a second and final estimate. If participants were in the no advice condition they saw “xxxx” in the place of the numbers. Participants did not receive feedback on the accuracy of their estimations during the experiment to inhibit learning about the rental market and the quality of advice. This allowed for the emulation of one-time decision making situations where people do not have the opportunity to track advice quality from repeated interactions with an advisor. Participants needed to rely upon the reputation of the advisor that was provided to them, either expert or novice.

Importantly, *all advice that participants received was the actual price of the apartment*. Thus, the only difference between the expert and novice conditions was the belief state of the participant that they were receiving advice from an expert or a novice.

After scanning, participants were asked to rate the value of each source of advice independently. They were asked, “Overall, how valuable was the advice you received from the expert/novice?” Participants were asked to respond on a Likert scale from 1 to 5, higher number denoting higher value. After responding to these questions, participants were debriefed about the experiment.

### Behavioral Analysis

**Figure 2:**
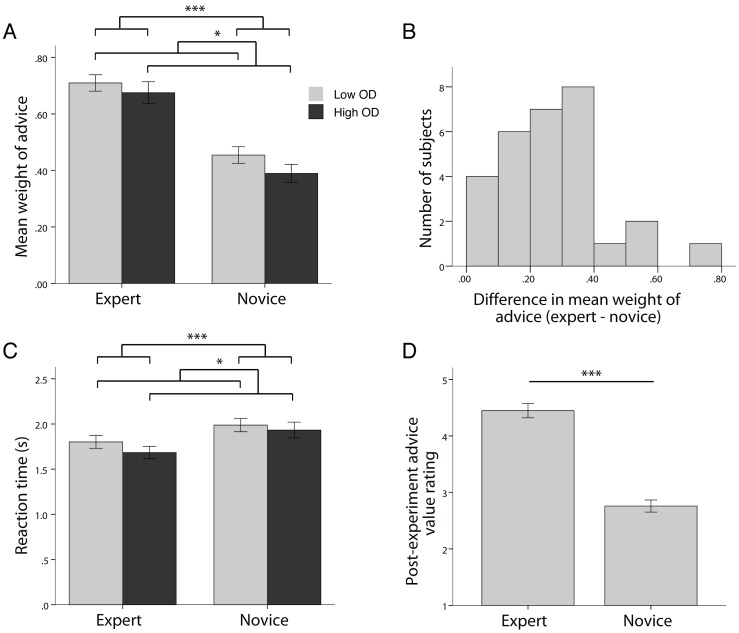
Behavioral data illustrating the utilization of advice. (A) Participants used expert advice more than novice advice (p<0.001). Important to note, all advice that participants received, from both experts and novices, was the actual price of the apartment (see Materials and Methods). Participants also used advice significantly more when the advice amount was close to their first estimate (low opinion difference) compared to when the advice was far from their first estimate (high opinion difference) (p = 0.017). (B) Histogram of individual differences in usage of advice with respect to expertise (mean expert WOA minus mean novice WOA). Participants demonstrated variability in their usage of advice from different sources. Notably however, all of the participants used expert advice qualitatively more than novice advice (no participants below zero). (C) Participants exhibited shorter reaction times when using expert advice than when using novice advice (p<0.001). They also responded more quickly when the opinion difference was high compared to when the opinion difference was low (p = 0.039). (D) After the experiment, participants rated the expert advice as being more valuable than the novice advice (scale from 1 =  low to 5 =  high; p<0.001). Error bars represent standard error of the mean. All analyses performed using repeated measures ANOVA unless otherwise indicated. OD  =  opinion difference.



All behavioral analyses were performed using repeated measures ANOVA unless otherwise indicated (see Figure 2). For each trial an “opinion difference” term was calculated as the absolute difference between the advice amount and the participant’s first estimate:

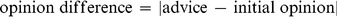
(1)For each participant, trials were classified according to the size of the opinion difference. Specifically, in each advice condition (expert and novice), trials were rank ordered by the size of the opinion difference and a median split was performed, separating trials into two groups, high opinion difference and low opinion difference.

The amount of advice utilization was quantified by calculating a weight of advice index (WOA) [2], [20]:
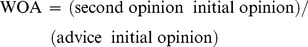
(2)


Importantly, we did not take the absolute value of this amount as previously done in the literature. A negative WOA indicates that participants moved away from the advice and we believe this is different behavior from moving towards the advice. Brain activity for these two behaviors would reflect this, thus we did not treat them equally in our neuroimaging analysis.

For an example of how this WOA calculation translates into an index of advice influence, assume two situations, both in which a participant’s initial opinion was 1000 Euro and the advice was 2000 Euro. In the first situation, at the time of receiving the advice, the participant may choose to adjust her estimate slightly to 1100 Euro. As a result, the weight of advice index would be calculated as 0.1. In the second situation, the participant may fully use the advice and adjust her estimate to 2000 Euro. As a result the calculated weight of advice index would be 1.

Trials in which participants displayed unusual reaction times were excluded from analysis. To do this, the log of all reaction times for the second estimate was calculated and times outside 2.5 standard deviations from a participant’s individual mean were selected for exclusion. The mean number of trials removed for each participant was 0.59 (SD = 0.63), the maximum removed for a participant was 2 trials. To assure that brain data were properly interpreted, other trials were removed from analysis. First, trials in which participants did not enter an estimate in either the first or second estimate were removed. Next, trials in which the WOA was zero were removed as well. This was done to achieve the goal of the study, which was to assess the influence of advice and not the decision of whether or not to use advice. If trials where the WOA was zero were included with trials in which participants decided to utilize advice to a certain degree, it would add a confound to the neuroimaging results because a different cognitive process may occur when the WOA is zero compared to even the smallest amount of advice utilization. Last, trials in which the WOA was greater than 1.3 were also removed. In these trials the participant’s second estimate was a large distance away from the advice amount. If these trials were left in the analysis they would be considered trials in which participants were highly influenced by the advice and this would be incorrect. To illustrate this point, please consider another two situations in which a participant’s first opinion was 1000 Euro and the advice was 2000 Euro. In the first situation, the participant may choose to adjust her opinion to 2100 Euro, and the resulting WOA would be 1.1. In the second situation, the participant may choose to adjust her opinion to 3100 Euro, and the resulting WOA would be 2.1. Although it is clear that in the second situation the participant’s second opinion is far from the advice and the participant was not as influenced by the advice compared to the first situation, this would not have been interpreted properly in the data because the 2.1 would have been coded as a trial where the advice was more influential than the 1.1. In order to stop this from happening, we removed all trials where the WOA was above 1.3. To summarize, for our analysis we excluded unanswered trials, trials with aberrant reaction times, and answered trials that had a WOA of zero or a WOA above 1.3. As a reminder, we included negative WOA trials (see above). Overall, this resulted in an average of 59.6 trials (SD = 11.6) remaining from the initial 80 trials per participant in the expert and novice conditions combined.

### fMRI Data Acquisition

Scanning was performed at the Dahlem Institute for Neuroimaging of Emotion at the Freie Universität Berlin, Germany using a 3T Siemens Trio scanner (Siemens Healthcare Diagnostics GmbH) and Siemens head coil. Stimuli were presented using the Cogent 2000 toolbox (http://www.vislab.ucl.ac.uk/cogent.php) for MATLAB (The Mathworks Inc.) on LCD-goggles (Resonance Technology Inc.). Anatomical images were acquired using a T1-weighted MPRage protocol (256×256 matrix, 176 sagittal slices of 1 mm thickness). Fieldmaps were acquired using a dual echo 2D gradient echo sequence with echos at 4.92 and 7.38 ms, and a repetition time of 488 ms. Functional images were acquired as echo-planar T2*-weighted images (repetition time = 2.0 s, echo time = 30 ms, matrix = 64×64, flip angle = 70°, field of view = 192 mm). A total of 37 contiguous oblique-axial slices (3×3×3 mm voxels) parallel to the anterior commissure-posterior commissure line were collected per volume. A total of 434 volumes were collected per experimental run, with 4 runs per participant.

### fMRI Data Analysis

FMRIB Software Library [21] (FSL, version 4.1.7) was used for fMRI data analysis on the High-Performance Computing system at Freie Universität Berlin (http://www.zedat.fu-berlin.de/Compute). Brain matter in the T1-weighted anatomical image was segmented from non-brain using a mesh deformation approach [22]. Functional data were preprocessed using FSL default options: motion correction was applied using rigid body registration to the central volume [23]; Gaussian spatial smoothing was applied with a full-width half-maximum of 6 mm; high-pass temporal filtering was applied using a Gaussian-weighted running lines filter, with a cut-off of 100 seconds. Susceptibility-related distortions were corrected as far as possible using FSL fieldmap correction routines [24].

To address our hypotheses concerning the valuation of advice, the assessment of the opinion difference, and a region of the brain representing the behavioral influence of advice, a general linear model was fit to the data with the following 14 regressors:

R1. For the periods of first estimation in the task.

**Figure 3:**
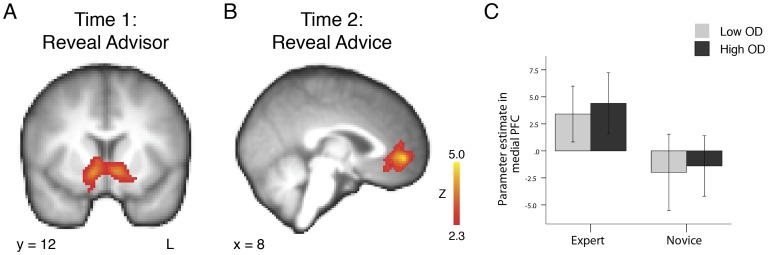
Brain regions showing a main effect between the expert and novice condition. (A) When contrasting expert > novice at Time 1, participants showed greater changes in BOLD signal in the ventral striatum upon discovering that their advisor will be an expert compared to discovering that their advisor will be a novice. (B) When contrasting expert > novice at Time 2, participants showed greater changes in BOLD signal in the medial prefrontal cortex when using advice from experts compared to using advice from novices. (C) Parameter estimates in the medial prefrontal cortex at Time 2. Bars indicate standard error of the mean. BOLD activation maps thresholded at Z >2.3, p<0.05, cluster corrected. L  =  left, OD  =  opinion difference, PFC  =  prefrontal cortex.
R4. It was revealed that advice would not be given.

For the periods when the advisor was revealed in the task (Time 1):

R2. An expert advisor was revealed.
R3. A novice advisor was revealed.
(R2 & R3 were used in the contrast for Figure 3A)

**Figure 4:**
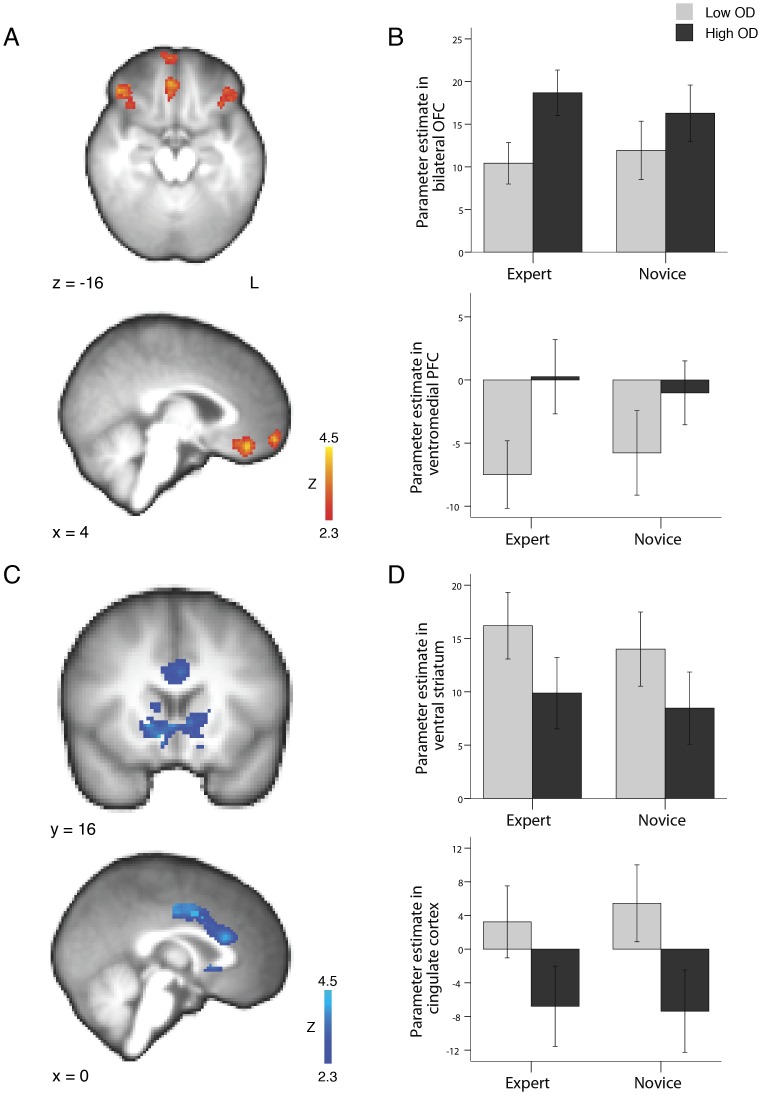
Brain regions demonstrating differential activity due to size of opinion difference upon revealing the advice (Time 2). (A) When contrasting high opinion difference trials > low opinion difference trials, changes in BOLD signal were greater in the lateral orbitofrontal cortex and the ventromedial prefrontal cortex. (C) When contrasting low opinion difference trials > high opinion difference trials, changes in BOLD signal were greater in the ventral striatum and the anterior cingulate cortex. (B,D) Parameter estimates in these regions. Bars indicate standard error of the mean. BOLD activation maps significant at p<0.01, FDR corrected within *a priori* defined areas (see Materials and Methods). L  =  left, OD  =  opinion difference, OFC  =  orbitofrontal cortex.

**Figure 5:**
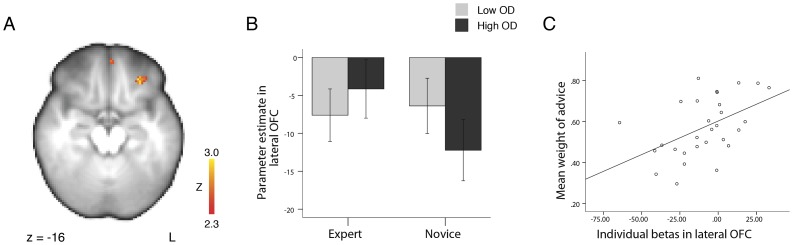
Interaction effect between expertise and size of opinion difference when advice was revealed (Time 2) in left lateral orbitofrontal cortex. (A) BOLD activity in an interaction contrast ((expert high opinion difference trials > expert low opinion difference trials) > (novice high opinion difference trials > novice low opinion difference trials)) revealed a significant interaction effect in the left lateral orbitofrontal cortex. BOLD activation map is significant at p<0.01, FDR corrected within *a priori* defined areas (see Materials and Methods). (B) Parameter estimates in the left lateral orbitofrontal cortex. Bars indicate standard error of the mean. (C) The mean activation across all advice trials in this lateral orbitofrontal region predicts the mean weight of advice over all advice trials for individual participants (Pearson’s r = 0.488, p = 0.007). L  =  left, OD  =  opinion difference.
R9. Participants received expert advice, modulated by the WOA for that trial. This regressor was orthogonalized to R5 and R6.
R10. Participants received novice advice, modulated by the WOA for that trial. This regressor was orthogonalized to R7 and R8.
R11. Participants made a second estimation without receiving advice.
R12. Participants received advice but did not use it (WOA = 0).

For the periods of second estimation in the task (Time 2):

R5. Participants received expert advice with a low opinion difference.
R6. Participants received expert advice with a high opinion difference.
R7. Participants received novice advice with a low opinion difference.
R8. Participants received novice advice with a high opinion difference.
(R5–R8 were used in the contrasts for Figures 3B,C and Figures 4 & 5)

Finally, two nuisance regressors captured:

R13. Subjects’ button presses to model motor activity.
R14. Error trials (see exclusions in Behavioral Analysis).

Durations of stimuli were computed by calculating actual on-screen time of stimuli (8.5 s for first estimate, 2.5 s for the revealing of the advisor, and 7 s for second estimate). The duration of motor activity was calculated from the first button press to the last. All regressors were convolved with the FSL default (gamma) hemodynamic response function. To note, our model contains two regressors at the time point where participants utilized advice (Time 2) that were modulated by the weight of advice on a trial-by-trial basis (R9, R10). R9 modeled trials in which participants received expert advice, and R10 modeled trials in which participants received novice advice. These regressors were orthogonalized with respect to the main effect regressors (see list above). The goal of including these regressors was to capture any additional, specifically linear parametric variance that was not already modeled in the unmodulated regressors. Analysis with these modulated regressors did not yield significant, reportable results. Individual contrast images were computed and taken to a group-level mixed-effect analysis using voxel-wise one-sample t-tests (see below).

To address our first hypothesis, to reveal BOLD signal changes representing the valuation of advice from different sources, we contrasted regressors at the time point (Time 1) when participants discovered they would be receiving expert or novice advice (R2> R3; see Figure 3A). Furthermore, to determine brain regions involved in utilizing expert or novice advice, we contrasted regressors at the time point (Time 2) when participants utilized advice and grouped the regressors by expertise level (R5+ R6> R7+ R8; see Figure 3B). Z-statistic images were thresholded with default FSL cluster correction for multiple comparisons with a minimum Z-score set at 2.3 and a significance level set at p<0.05. Parameter estimates were extracted by contrasting indicated regressors against baseline (R5, R6, R7, R8; see Figure 3C).

To address our second hypothesis, to reveal BOLD signal changes representing the comparison of the participants’ initial opinion and the advice, we contrasted regressors at the time point (Time 2) when participants received advice and grouped the regressors by the size of the opinion difference (R6+ R8> R5+ R7; and reverse contrast; see Figures 4A, C). Importantly, advice is commonly used when making goal-oriented decisions where people try to maximize reward and/or minimize punishment. In our task, we emulated this by providing a monetary incentive to participants, where they believed they would be rewarded for the accuracy of their estimations. Therefore, when participants made their first estimate and then discovered the advice amount, they calculated an opinion difference that was directly related to the probability that they would receive a reward, depending on how much they valued the advice source. For example, if the participant valued the advice source and found out there was a high opinion difference, then they would think their estimate needs revision in order to obtain a reward. This demonstrates the direct relation of the opinion difference to reward. Furthermore, prior behavioral research has shown that advice discounting is affected by monetary reward [3], [20], [25]. If the size of the monetary reward affects the weight of advice, it could be that calculation of the opinion difference, which occurs during the only instance that participants receive information from their advisors, is reflected in reward areas. Therefore, due to the above two lines of reasoning, we hypothesized that the opinion difference would be calculated by brain regions that have previously been established to be reward-sensitive, such as the ventral striatum, amygdala, anterior cingulate gyrus, ventromedial prefrontal cortex and the orbitofrontal cortex [5]–[7], [9]–[11]. For analysis of neuroimaging data related to the opinion difference, we created a region of interest mask of these reward-sensitive areas. Regions were defined by the Harvard-Oxford anatomical atlas (75% minimum probability threshold, 2 mm resolution): bilateral nucleus accumbens, bilateral caudate, bilateral putamen, bilateral amygdala, anterior cingulate cortex, orbitofrontal cortex, and medial prefrontal cortex. These regions in the atlas did not cover all reward-related areas and gaps remained at the anterior/medial prefrontal cortex and the ventral/lateral orbitofrontal cortex. Thus, these areas were drawn in manually from the ventral paracingulate gyrus and the ventral/anterior frontal pole regions in the atlas. The resulting mask was then smoothed using a mean-filtered kernel of 3.5 mm. To reveal BOLD signal changes concerning the opinion difference using this region of interest mask, Z-statistic images were thresholded using false discovery rate (FDR) correction for multiple comparisons with a significance level set conservatively at p<0.01. Parameter estimates were extracted by contrasting indicated regressors against baseline (R5, R6, R7, R8; see Figures 4B, D).

To address our third hypothesis, that there is a region of the brain where neural activity represents the behavioral influence of advice, we performed an interaction contrast at the time point (Time 2) when participants received advice ((R6> R5) > (R8> R7); see Figure 5A). Because of the involvement of the opinion difference in the contrast, we used the above-described region of interest mask to reveal BOLD signal changes. Z-statistic images were thresholded using false discovery rate (FDR) correction for multiple comparisons with a significance level set conservatively at p<0.01. Parameter estimates were extracted by contrasting indicated regressors against baseline (R5, R6, R7, R8; see Figure 5B).

## Results

### Behavioral Results

Behavioral data revealed that participants used expert advice more than novice advice as demonstrated by the weight of advice index (F_(1,28)_ = 77.531, p<0.001; Figure 2A). Weight of advice also differed as a function of opinion difference, the distance between a participant’s initial opinion and the advice amount. When participants experienced a low opinion difference they used advice more than when they experienced a high opinion difference (F_(1,28)_ = 6.386, p = 0.017; Figure 2A). Participants also displayed individual differences in how they utilized advice from experts and novices (Figure 2B). Some participants used a similar amount of advice from both expert and novice sources (none used more novice than expert), while others displayed a greater use of expert advice compared to novice advice. Importantly, we wanted to keep performance constant over the course of the experiment and therefore did not give feedback to participants about the actual price of the apartments. Indeed, participants did not improve the accuracy of their opinions; the overall difference between the real price and participants’ first opinions did not differ between the first and second half of the experiment (paired t_(28)_ = 0.309, p = 0.759).

We also analyzed participants’ reaction times when utilizing advice (Figure 2C). Participants responded more quickly when using expert advice than when using novice advice (F_(1,28)_ = 24.754, p<0.001). In addition, they took significantly longer to respond when the opinion difference was low (F_(1,28)_ = 4.699, p = 0.039).

After the experiment, participants were asked to rate the value of each type of advice on a Likert scale from 1 to 5, with the higher number indicating a higher value. The value of the expert advice was rated as greater than the value of the novice advice (paired t_(28)_ = 10.217, p<0.001; Figure 2D).

### Neuroimaging Results

#### The value of advice

We hypothesized that the same areas which represent value and reward expectation when receiving money and objects, such as the ventral striatum [6], also represent value when receiving advice. To address this, we analyzed neural activity when participants discovered that advice would be coming from an expert or a novice (Time 1). In the expert > novice contrast participants showed greater blood oxygenation level-dependent (BOLD) signal changes in the ventral striatum (peak voxel MNI coordinates: 10, 12, −8; max Z = 4.52; p<0.05, cluster corrected; Figure 3A). See Table 1 for a complete list of brain regions demonstrating a significant activation in this contrast, and the novice > expert reverse contrast.

**Table 1:** Significant activation clusters for expertise contrasts at Time 1 when participants discovered whom their advisor will be.

	MNI Coordinates				
Region	x	y	z	Cluster size	Peak z
Expert > Novice					
L/R Ventral striatum	10	12	−8	1478	4.52
R Occipital cortex	16	−98	4	1379	6.02
L Occipital cortex	−22	−100	4	1368	5.46
Novice > Expert					
R Angular gyrus	42	−58	20	1058	4.45
L/R Precuneus	10	−50	42	686	3.66

Z >2.3, p<0.05, cluster corrected. L, Left; R, Right.

Although not directly relevant to our research question, we also compared neural activity in the control condition where participants found out they would not be receiving advice to the experimental condition where they found out they would be receiving advice (main effect of advice across the expert and novice conditions). See Figure S1 and Table S1 for results.

#### Expert versus novice advice utilization

To examine brain activity associated with utilizing advice from sources with different levels of expertise, we analyzed brain activity when participants received the advice (Time 2). In the expert > novice contrast participants showed greater changes in BOLD signal in three regions (p<0.05, cluster corrected): the medial prefrontal cortex (0, 50, −2; max Z = 4.63; Figure 3B), the left superior parietal lobule (−30, −56, 50; max Z = 3.69; Table 2) and the left inferior temporal gyrus (−52, −48, −14; max Z = 4.07). There were no significant activations in the novice > expert contrast using the same strict thresholding procedure.

**Table 2:** Significant activation clusters for expertise contrasts at Time 2 when participants used advice.

	MNI Coordinates				
Region	x	y	z	Cluster size	Peak z
Expert > Novice					
L Superior parietal lobule	−30	−56	50	1710	3.69
L Inferior temporal gyrus	−52	−48	−14	1428	4.07
L/R Paracingulate/Medial PFC	0	50	−2	1175	4.63
Novice > Expert					
None					

Z >2.3, p<0.05, cluster corrected. PFC, prefrontal cortex.

In addition, we contrasted the control condition where participants did not receive advice and re-evaluated their opinion with the experimental condition where participants used advice (both expert and novice conditions). See Figure S2 and Table S2 for results.

#### The opinion difference

We hypothesized that the opinion difference would be represented in previously established reward-sensitive regions. To address this, we analyzed neural activity when participants received advice (Time 2) with respect to the size of the opinion difference. In the high opinion difference > low opinion difference contrast, three regions displayed a greater change in BOLD signal (p<0.01, FDR corrected within *a priori* defined reward-sensitive areas – see Materials and Methods; Figure 4A): the bilateral orbitofrontal cortex (right: 46, 36, −16; max Z = 3.54; left: −34, 22, −20; max Z = 3.27), the ventromedial prefrontal cortex (6, 40, −18; max Z = 3.68) and the medial frontal pole (4, 62, −14; max Z = 3.59; Table 3). In the low opinion difference > high opinion difference contrast, there was a greater change in BOLD signal in four regions (p<0.01, FDR corrected within *a priori* defined areas; Figure 4C): the ventral striatum (10, 16, −8; max Z = 4.69), the anterior cingulate cortex (−2, −2, 42; max Z = 3.58), the bilateral putamen (right: 30, −14, 6; max Z = 3.56; left: −30, −4, 6; max Z = 3.96; Table 3) and the left lateral orbitofrontal cortex (−22, 46, −16; max Z = 3.2).

**Table 3:** Significant activation clusters for opinion difference contrasts at Time 2 when participants received advice.

	MNI Coordinates				
Region	x	y	z	Cluster size	Peak z
High OD > Low OD					
L/R Ventromedial frontal cortex	6	40	−18	224	3.68
R Lateral orbitofrontal cortex	46	36	−16	208	3.54
L Lateral orbitofrontal cortex	−34	22	−20	180	3.27
L/R Frontal pole	4	62	−14	133	3.59
Low OD > High OD					
L/R Anterior cingulate gyrus	−2	−2	42	1138	3.58
L/R Ventral striatum	10	16	−8	638	4.69
L Putamen	−30	−4	6	269	3.96
R Putamen	30	−14	6	206	3.56
L Lateral orbitofrontal cortex	−22	46	−16	52	3.2

p<0.01, FDR corrected within *a priori* defined areas (see Materials and Methods). Clusters >40 voxels. OD, opinion difference.

#### The influence of advice

We tested the hypothesis that changes in BOLD signal in areas integrating both the expertise level of the advisor and the opinion difference would correlate with the behavioral influence of advice. We first computed an interaction contrast, (expert high opinion difference trials > expert low opinion difference trials) > (novice high opinion difference trials > novice low opinion difference trials), when participants utilized advice (Time 2) to find these integration areas. This analysis reveals any brain region in which the BOLD signal change in response to a change in one factor (eg. opinion difference) depended upon the other factor (eg. expertise level of the advisor). This analysis revealed a significant interaction effect in two regions (p<0.01, FDR corrected within *a priori* defined areas; Figure 5A and Table 4): the left orbitofrontal cortex (−30, 38, −14; max Z = 3.02) and the ventromedial prefrontal cortex (0, 56, −22; max Z = 2.85). There were no significant interactions in the reverse contrast.

**Table 4:** Significant activation clusters for opinion difference interaction contrasts at Time 2 when participants received advice.

	MNI Coordinates				
Region	x	y	z	Cluster size	Peak z
(Expert high OD > Expert low OD) > (Novice high OD > Novice low OD)					
L/R Ventromedial frontal cortex	0	56	−22	49	2.85
L/R Lateral orbitofrontal cortex	−30	38	−14	48	3.02
(Expert low OD > Expert high OD) > (Novice low OD > Novice high OD)					
None					

p<0.01, FDR corrected within *a priori* defined areas (see Materials and Methods). Clusters >40 voxels. OD, opinion difference.

We had hypothesized that the activity in areas that demonstrate an interaction between the expertise level of an advisor and the opinion difference would correlate with the individual weight of advice. Thus, for each participant, while they made their second estimate, we extracted the parameter estimate across all advice conditions against baseline (expert high opinion difference trials + expert low opinion difference trials + novice high opinion difference trials + novice low opinion difference trials)/4 from the two regions demonstrating the interaction effect. We then performed a correlation analysis with the parameter estimate and the mean weight of advice across all trials for each participant. The changes in activity in the lateral orbitofrontal cortex correlated with the mean weight of advice across participants (Pearson’s r = 0.488, p = 0.007; Figure 5C). The changes in activity in the ventromedial prefrontal cortex did not correlate significantly with the mean weight of advice (Pearson’s r = −0.186, p = 0.333). To note, there was no correlation between the interaction parameter estimate and the mean weight of advice across participants in either the lateral orbitofrontal cortex (Pearson’s r = 0.103, p = 0.596) or the ventromedial prefrontal cortex (Pearson’s r = −0.219, p = 0.253).

## Discussion

In the present study, we designed a task to emulate real world decision making situations where people form an initial opinion, discover they will be receiving advice (along with the expertise level of their advisor), receive advice and then adjust their opinion to make a final decision. To better understand the neurocognitive processes involved in these types of decisions, we varied the expertise level of the advisor and ensured that participants experienced variations in the size of the opinion difference on a trial-by-trial basis. This resulted in participants exhibiting a behavioral change that we quantified with the weight of advice index.

Our behavioral results demonstrate that participants valued expert advice more than novice advice, as indicated in the post-experiment questionnaire. Participants used advice from both groups of advisors, but they used advice from experts more than advice from novices. This result replicates previous behavioral research demonstrating that people use more advice when it comes from experts [2]–[4]. Before participants received the actual advice, they also displayed greater changes in BOLD signal in the ventral striatum when they discovered that they would be receiving expert advice compared to novice advice. This result agrees with previous research demonstrating that activity in the ventral striatum tracks value through reward anticipation [6], [26], [27]. People may value expert advice more because they believe it will enable them to make better decisions with higher value outcomes, even before they receive a specific recommendation. Brain activity at the time of the utilization of expert and novice advice supports this view. Participants demonstrated greater increases in BOLD signal in the medial prefrontal cortex when utilizing expert advice. This result is in line with previous research demonstrating that activity in this region positively correlates with the value of a chosen option when choosing between options; the higher the expected value of the choice, the higher the activity [28]–[31].

We theorized that the distance between a person’s initial opinion and the advisor’s opinion would affect advice utilization. Our behavioral results demonstrate that participants used advice more when the distance between their first estimate and the advice was low. A recent behavioral study which asked people to estimate historical dates, such as the year the Suez Canal first opened, while either receiving or not receiving advice, found the same result [8]. To note, the size of the difference in the weight of advice index between the high and low opinion difference conditions in this study was comparable to our study, 0.08 and 0.07, respectively (in both studies the differences are small yet significant). Our neuroimaging results show that the opinion difference is represented in brain regions previously indicated to be involved in reward processing. When the opinion difference was high, an increased BOLD signal was observed in the lateral orbitofrontal cortex and the ventromedial prefrontal cortex. When the opinion difference was low, an increased BOLD signal was observed in the ventral striatum and the anterior cingulate cortex. In low opinion difference trials participants found out that someone else’s opinion was similar to their own. In other studies the ventral striatum has also been shown to be more active when people found out another’s opinion was similar to their own [15], [16]. With the ventral striatum being firmly established as a reward-related brain area [32], it is possible that people experience a reward when they find out that their opinion is close to an advisor’s opinion, i.e. when a person agrees with them.

With respect to our anterior cingulate cortex result, it has been well documented that this area is involved in computing rewards during behavioral tasks [10]. Similar to the ventral striatum, our finding that anterior cingulate cortex is more active in low opinion difference trials can be interpreted with regard to this reward literature, although it may be somewhat surprising when considering its role in other previous literature on conflict monitoring and cognitive control [33], [34]. Importantly, our reaction time data show that participants took longer to respond when their initial opinion was close to the advisor’s estimate. This difference was small yet significant. We theorize that when the opinion difference is low, a slightly more “fine-grained” estimation ensues resulting in a slightly longer reaction time. Alternatively, it could be that the participants are choosing between two options, their first opinion versus the advice, and in the low opinion difference condition, the two options are closer together and thus present a slightly more difficult decision (although previous behavioral research shows that people who receive advice tend to average opinions rather than choose between an initial opinion and an advisor’s [35]). Either way, our reaction time data suggest a greater amount of information processing when encountering low opinion differences and agrees with the previous literature on the role of the anterior cingulate cortex [33], [34], [36]–[38].

We identified a brain region that represents the behavioral influence of advice by requiring that this region fulfill two conditions. First, when the participant utilizes advice, the expertise of the advisor and the size of the opinion difference should interact in this area. Second, the activity in this region should correlate with individual differences in advice utilization across participants. We found that activity in the left lateral orbitofrontal cortex fulfilled these requirements. Specifically, we observed this correlation with the average parameter estimate across all advice conditions against baseline. Thus, our data demonstrate that, across individuals, the greater the average BOLD signal change in the left lateral orbitofrontal cortex during decision making, the greater the influence of advice.

Similar to the present study on explicit advice, certain types of implicit influence by celebrities or group opinion have previously been investigated. For example, activity in the anterior cingulate cortex was demonstrated to correlate with the perceived degree of expertise a celebrity has regarding a product [18]. It was shown that the next day after viewing a celebrity paired with a product, the greater the perceived expertise of the celebrity, the greater the intention to purchase the product and the greater the memory for the product. This study provided evidence for the implicit influence of expertise on decision making, and although in the present study we focused on the explicit influence of expertise, our results agree with their behavioral findings, showing that people are more influenced by individuals whom they perceive to have more expertise. Furthermore, conformity to group opinion has previously been shown to recruit the intraparietal sulcus, temporoparietal junction, insular cortex, anterior cingulate, ventral striatum and the lateral orbitofrontal cortex [15]–[17], [39], [40]. Importantly, in the most recent study by Campbell-Meiklejohn et al., behavioral conformity was correlated with brain structure to reveal a peak voxel in the left lateral orbitofrontal cortex (−33, 28, −16) that is very near to the peak voxel revealed by our functional interaction contrast (−30, 38, −14). Thus, our results concerning advice utilization, taken together with this recent publication on conformity, strongly suggest a role for the lateral orbitofrontal cortex in the computation of social influence.

Previous neuroimaging research has investigated aspects of advice taking that are different from the present study. Neural correlates for receiving advice, compared to not receiving advice, have been demonstrated in the dorsomedial prefrontal cortex and the temporoparietal junction [13]. Furthermore, when making repeated decisions with the same advisor and receiving feedback on decision outcomes, the dorsomedial prefrontal cortex and the temporoparietal junction are active during the outcome period [12]. In this second study, these regions computed a social prediction error allowing a person to learn the trustworthiness of their advisor. Finally, it has been demonstrated that the septal area implements an “outcome-bonus” signal upon receiving feedback after choices made under the influence of advice [14]. The septal area demonstrated a greater signal change after both positive and negative feedback from recommended choices compared to non-recommended choices. The current study did not demonstrate involvement of similar brain regions. However, this is not surprising because we focused on the differences between using expert and novice advice, and not the differences between making decisions with or without advice. Furthermore, we investigated brain activity at the time participants received advice and related it to behavioral change via the weight of advice index. Examining brain activity at the time we receive and utilize advice and relating it to the behavioral change caused by the advice is crucial to understanding how we integrate advice into the decision making process.

In conclusion, with the present report, we demonstrate how people use advice when making decisions. We show that advice-taking consists of three neurocognitive processes: the valuation of advice, the assessment of the opinion difference, and the process of combining valuation and the opinion difference resulting in actual advice utilization. This last process was shown to occur in the left lateral orbitofrontal cortex, where the average activity correlates with the mean use of advice across participants. This result establishes the lateral orbitofrontal cortex as a region of the brain responsible for the behavioral influence of advice. As a whole, our findings provide neural evidence for how advice engenders behavioral change during the decision making process, and advance the overall understanding of how humans use advice.

## Supporting Information

Figure S1 **Brain regions showing a main effect between the advice and no advice conditions when participants discovered whom their advisor will be (Time 1).** Advice includes both the expert and novice conditions. (A) When contrasting advice > no advice, participants showed greater changes in BOLD signal in the ventral striatum and medial prefrontal cortex. (B) When contrasting no advice > advice, participants showed greater changes in BOLD signal in the right caudate and dorsolateral prefrontal cortex. BOLD activation maps thresholded at Z >3.7, p<0.05, cluster corrected. L = left.
(TIF)

Figure S2 **Brain regions showing a main effect between the advice and no advice conditions when participants received advice (Time 2).** Advice includes both the expert and novice conditions. (A) When contrasting advice > no advice, participants showed greater changes in BOLD signal in the caudate and intraparietal sulcus. (B) When contrasting no advice > advice, participants showed greater changes in BOLD signal in the insula and inferior parietal lobule. BOLD activation maps thresholded at Z >3.7, *P*<0.05, cluster corrected. L = left.
(TIF)

Table S1 **Significant activation clusters at Time 1 when participants discovered if they would be receiving advice (expert & novice) or not receiving advice.** Z >3.7, p<0.05, cluster corrected. L, Left; R, Right; dlPFC, dorsolateral prefrontal cortex.
(DOCX)

Table S2 **Significant activation clusters at Time 2 when participants either received advice (expert & novice) or did not receive advice and re-evaluated their opinion.** Z >3.7, p<0.05, cluster corrected.
(DOCX)
