# Supplementary figures and images for: How Expert Advice Influences Decision Making
Source: PLoS One. 2012 Nov 21;7(11):e49748. doi: 10.1371/journal.pone.0049748 (PMC3504100; doi:10.1371/journal.pone.0049748)

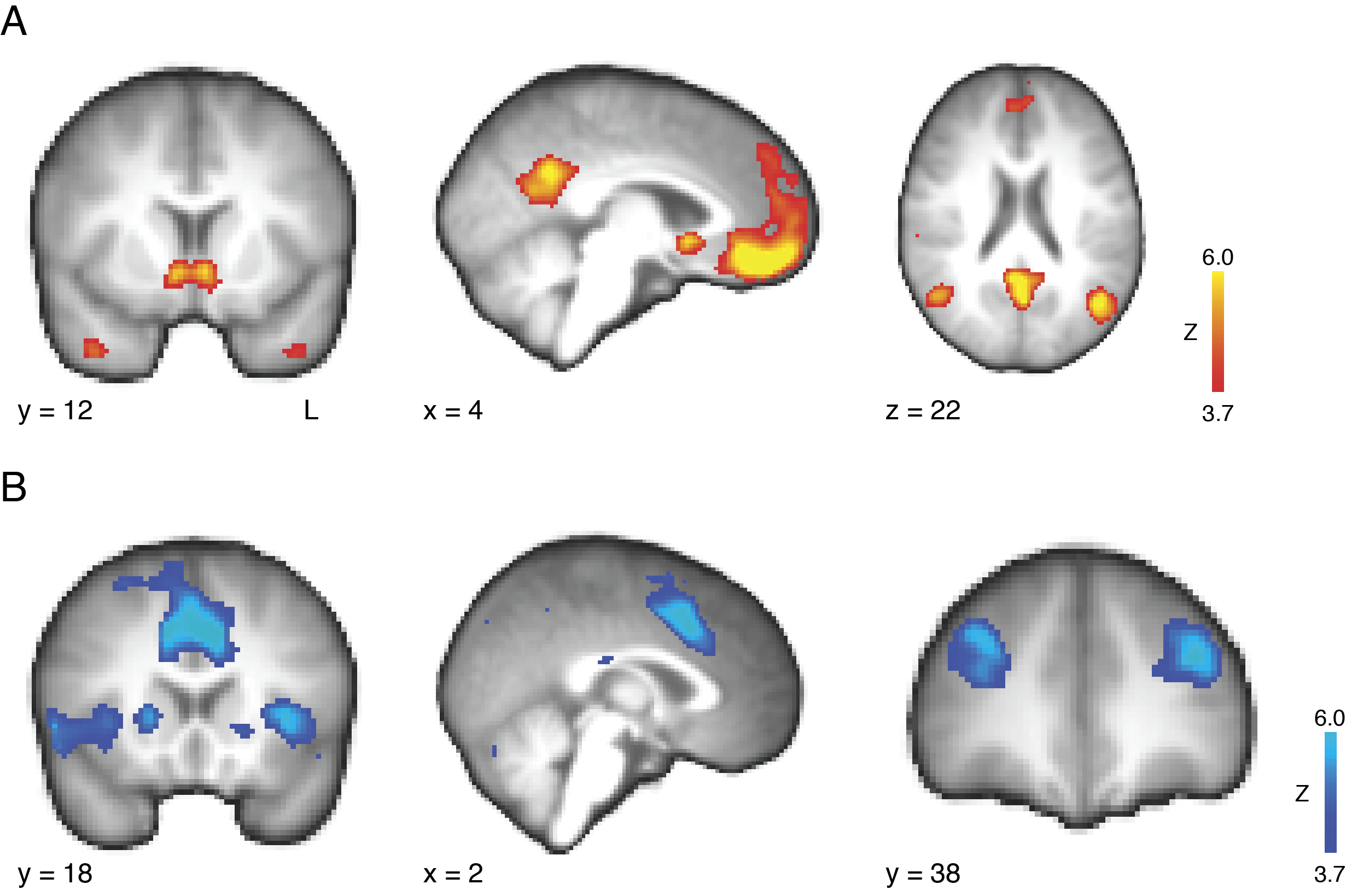

Supplement: Figure S1 — Brain regions showing a main effect between the advice and no advice conditions when participants discovered whom their advisor will be (Time 1). Advice includes both the expert and novice conditions. (A) When contrasting advice > no advice, participants showed greater changes in BOLD signal in the ventral striatum and medial prefrontal cortex. (B) When contrasting no advice > advice, participants showed greater changes in BOLD signal in the right caudate and dorsolateral prefrontal cortex. BOLD activation maps thresholded at Z >3.7, p<0.05, cluster corrected. L = left. (TIF) [file pone.0049748.s001.tif]

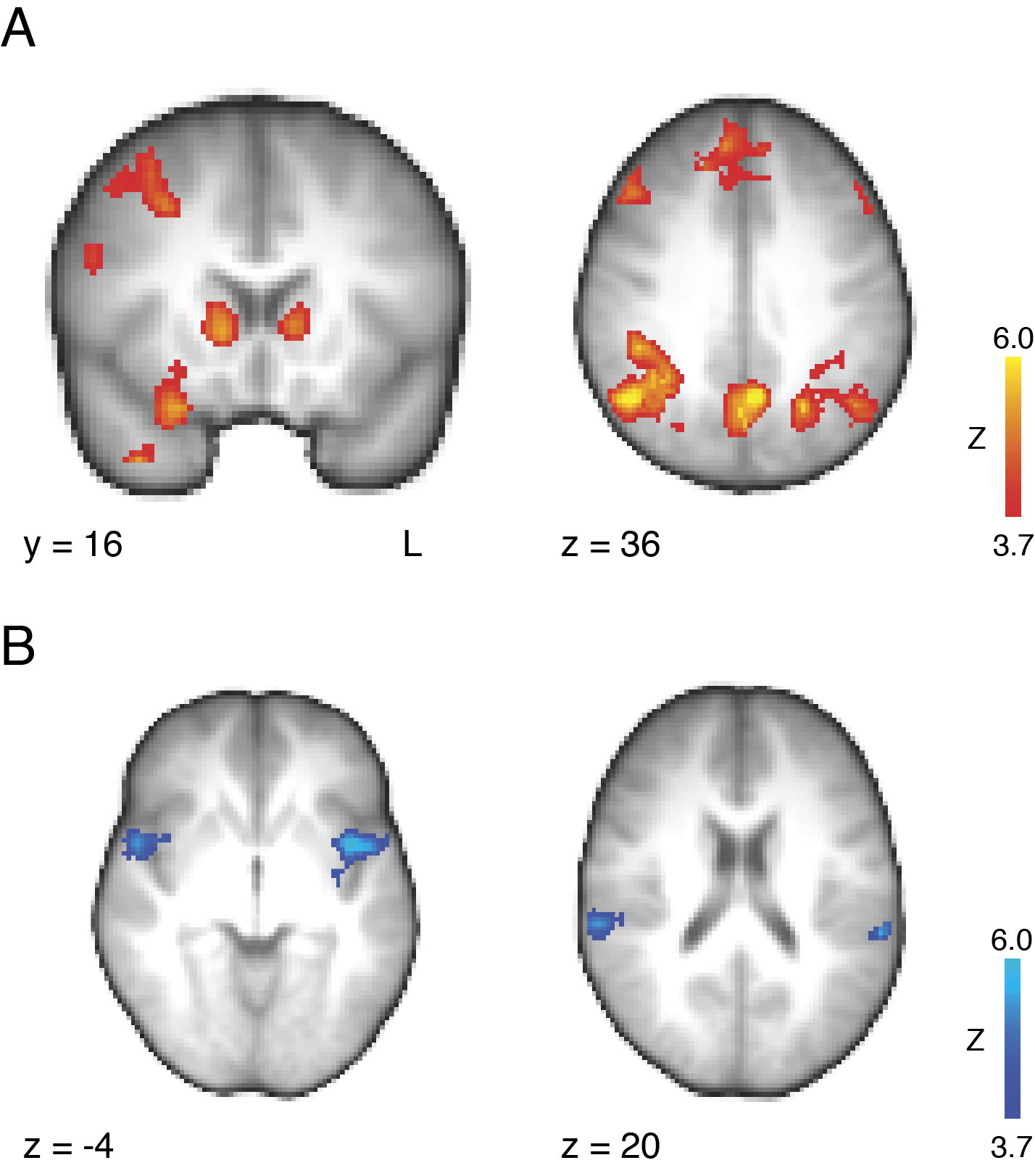

Supplement: Figure S2 — Brain regions showing a main effect between the advice and no advice conditions when participants received advice (Time 2). Advice includes both the expert and novice conditions. (A) When contrasting advice > no advice, participants showed greater changes in BOLD signal in the caudate and intraparietal sulcus. (B) When contrasting no advice > advice, participants showed greater changes in BOLD signal in the insula and inferior parietal lobule. BOLD activation maps thresholded at Z >3.7, P<0.05, cluster corrected. L = left. (TIF) [file pone.0049748.s002.tif]
