# Supplementary material for: How Expert Advice Influences Decision Making
Source: PLoS One. 2012 Nov 21;7(11):e49748. doi: 10.1371/journal.pone.0049748 (PMC3504100; doi:10.1371/journal.pone.0049748)
Supplement: Table S1 — Significant activation clusters at Time 1 when participants discovered if they would be receiving advice (expert & novice) or not receiving advice. Z >3.7, p<0.05, cluster corrected. L, Left; R, Right; dlPFC, dorsolateral prefrontal cortex. (DOCX) [file pone.0049748.s003.docx]

|  | | MNI Coordinates | | |  |  |
| --- | --- | --- | --- | --- | --- | --- |
| Region | | x | y | z | Cluster size | Peak z |
| Advice > No Advice | |  |  |  |  |  |
|  | Medial prefrontal cortex | -4 | 50 | -18 | 3827 | 7.69 |
|  | Precuneus | 0 | -56 | 30 | 1018 | 6.30 |
|  | L Lateral occipital cortex | -44 | -64 | 22 | 509 | 6.33 |
|  | Ventral striatum | -4 | 12 | -6 | 334 | 5.64 |
|  | L Middle temporal gyrus | -58 | -10 | -20 | 226 | 5.95 |
|  | R Lateral occipital cortex | 50 | -60 | 20 | 217 | 5.38 |
|  | R Temporal pole | 40 | 14 | -38 | 104 | 4.85 |
|  | L Temporal pole | -42 | 10 | -40 | 65 | 4.3 |
| No Advice > Advice | |  |  |  |  |  |
|  | Superior frontal gyrus | 24 | 4 | 56 | 5609 | 6.81 |
|  | Precuneus | 18 | -64 | 38 | 3604 | 5.62 |
|  | L Frontal pole/dlPFC | -38 | 38 | 26 | 1571 | 5.96 |
|  | R Frontal pole/dlPFC | 34 | 42 | 32 | 1226 | 5.89 |
|  | L Cerebellum | -40 | -58 | -34 | 869 | 5.61 |
|  | R Cerebellum | 44 | -70 | -28 | 815 | 5.83 |
|  | L Insular cortex | -36 | 20 | 2 | 619 | 5.77 |
|  | R Caudate | 18 | 16 | 6 | 167 | 5.30 |
|  | R Cerebellum | 38 | -62 | -54 | 77 | 4.47 |
|  | L Cerebellum | -22 | -78 | -46 | 68 | 4.62 |
