# Supplementary material for: How Expert Advice Influences Decision Making
Source: PLoS One. 2012 Nov 21;7(11):e49748. doi: 10.1371/journal.pone.0049748 (PMC3504100; doi:10.1371/journal.pone.0049748)
Supplement: Table S2 — Significant activation clusters at Time 2 when participants either received advice (expert & novice) or did not receive advice and re-evaluated their opinion. Z >3.7, p<0.05, cluster corrected. (DOCX) [file pone.0049748.s004.docx]

|  | | MNI Coordinates | | |  |  |
| --- | --- | --- | --- | --- | --- | --- |
| Region | | x | y | z | Cluster size | Peak z |
| Advice > No Advice | |  |  |  |  |  |
|  | L Occipital cortex | -16 | -92 | -14 | 4144 | 6.51 |
|  | R Middle frontal gyrus | 50 | 26 | 32 | 4128 | 5.69 |
|  | R Anterior intraparietal sulcus | 34 | -52 | 40 | 2094 | 5.83 |
|  | R Occipital cortex | 20 | -94 | -10 | 1678 | 6.37 |
|  | L Anterior intraparietal sulcus | -24 | -62 | 38 | 1273 | 5.48 |
|  | R Lateral orbitofrontal cortex | 40 | 22 | -14 | 1040 | 5.32 |
|  | Precuneus | -4 | -58 | 36 | 689 | 5.63 |
|  | Cerebellum | -4 | -60 | -42 | 336 | 5.37 |
|  | R Inferior temporal gyrus | 58 | -34 | -16 | 334 | 5.32 |
|  | R Cerebellum | 30 | -74 | -38 | 167 | 5.14 |
|  | L Inferior temporal gyrus | -54 | -42 | -16 | 160 | 4.36 |
|  | R Caudate | 12 | 18 | 0 | 150 | 5.02 |
|  | L Superior frontal gyrus | -16 | 28 | 54 | 149 | 4.50 |
|  | L Middle frontal gyrus | -48 | 28 | 32 | 148 | 4.37 |
|  | L Middle temporal gyrus | -62 | -10 | -24 | 117 | 4.29 |
|  | R Middle temporal gyrus | 58 | -4 | -32 | 92 | 4.46 |
|  | L Caudate | -10 | 16 | 4 | 82 | 4.65 |
| No Advice > Advice | |  |  |  |  |  |
|  | L Insular cortex | -40 | 8 | 0 | 434 | 6.18 |
|  | R Planum polare | 54 | 6 | -4 | 199 | 5.17 |
|  | R Inferior parietal lobule | 62 | -30 | 22 | 198 | 5.24 |
|  | L Inferior parietal lobule | -60 | -34 | 20 | 87 | 5.29 |
